# Revisiting multi‐stage models for upstream technology adoption: Evidence from rapid generation advance in rice breeding

**DOI:** 10.1111/1477-9552.12450

**Published:** 2021-07-04

**Authors:** Bert Lenaerts, Yann de Mey, Matty Demont

**Affiliations:** ^1^ Centre for Environmental Sciences UHasselt Hasselt Belgium; ^2^ International Rice Research Institute Los Baños Philippines; ^3^ Division of Bioeconomics Department of Earth and Environmental Sciences KU Leuven Belgium; ^4^ Business Economics Group (BEC) Wageningen University & Research the Netherlands

**Keywords:** accelerated breeding, innovation system, rice, selection model, triple‐hurdle

## Abstract

Adoption of new plant varieties has played a significant role in eradicating global hunger. Previous research has mainly focused on farmer adoption and impact of new crop varieties, although upstream adoption of technologies in plant breeding can generate substantial multiplier effects on downstream impacts. This study moves upstream in the innovation system to generate policy advice on adoption and transfer of accelerated rice breeding technologies. More specifically, we assess the determinants of global adoption of rapid generation advance (RGA) through a sample of 158 rice breeders operating in various research institutes worldwide. Moving upstream in the innovation system has important theoretical and empirical implications due to the smaller number of decision‐making units in the adoption process and the increasing role of institutional and managerial factors that may overrule individual adoption motivations. We revisit multi‐stage models and devise the most robust estimation method that can be used in this situation. To generate insights on the impact of individual versus institutional adopter characteristics on upstream technology adoption, we juxtapose the response curves of the determinants of RGA adoption in rice breeding among alternative adoption stages, levels of conditionality and model specifications. Our findings confirm the importance of institutional and managerial factors and suggest that adoption and transfer of breeding technologies require breeding institutes to provide an enabling environment in which breeders are encouraged to take risks and are given sufficient freedom to experiment with and implement new technologies.

## 1 INTRODUCTION

Adoption of new crop varieties by farmers is one of the most widely studied topics in agricultural economics (Besley & Case, 1993; Feder et al., 1985; Kafle, 2010; Marra et al., 2003; Sunding & Zilberman, 2001). Although most adoption studies focus on farmers, farm‐level adoption of varieties crucially hinges on the quality and speed of plant breeding programmes upstream in the innovation system. Previous technology adoption studies have almost solely been focused on farmers. The question of technology adoption by crop breeders is mostly unaddressed in the present literature. For example, Rogers’ (2003) seminal work, ‘Diffusion of Innovations’, only mentions the word ‘breeder(s)’ twice in contrast to mentioning the word ‘farmer(s)’ no less than 84 times. Sunding and Zilberman’s (2001) handbook chapter on this topic does not mention breeders while referring to farmers 73 times. Similarly, a recent special issue in *Applied Economic Perspectives and Policy* on ‘Adoption of Agricultural Innovations’ focuses entirely on downstream farm‐level adoption, while upstream adoption is not addressed (Pannell & Zilberman, 2020).

Among agricultural scientists, breeding is considered a principal activity in improving agricultural performance (such as improving yield), especially in the face of emerging challenges (abiotic and biotic stresses). Breeding of new varieties was one of the cornerstones of the Green Revolution in wheat, rice, and maize (Evenson & Gollin, 2003) and even today, production increases can in no small degree be attributed to genetic innovation (Khush, 2005; Tester & Langridge, 2010). However, plant breeding is typically characterised by long production processes that dramatically reduce its responsiveness to unpredictable changes in crop production environments, climate or markets (Lenaerts et al., 2019a). Challenges such as increasing demand and climate change will determine the future state of food security and might even—if no action is taken—hold back or reverse progress toward a world without hunger (Beddington, 2010; Wheeler & Von Braun, 2013). Due to their interaction and mutual reinforcement, these factors are expected to amplify the overall burden of food insecurity and the consequent need to transform food systems (Reardon & Timmer, 2014). Therefore, rapid breeding and cultivar delivery systems can be an important driver of climate change adaptation and global food security (Atlin et al., 2017). In many cases, however, especially in the developing world, development, as well as dissemination and adoption, of new technologies are severely hampered, limiting their potential to improve food security.

The International Rice Research Institute (IRRI)—a leading global public breeding institute—is currently accelerating its breeding programme through a method named ‘rapid generation advance’ (RGA) (Atlin et al., 2017; Collard et al., 2019). Accelerated breeding has the potential to increase adoption benefits due to earlier release (Lenaerts et al., 2018b), reduced operational costs (Collard et al., 2017) and an overall increase in breeding efficiency (Bonnecarrere et al., 2019). Despite being developed in the 1960s, this technology remains vastly underused in breeding institutes (Lenaerts et al., 2018a). Understanding why accelerated breeding methods such as RGA remain underused is an essential step in accelerating global crop breeding through technology transfer, especially in public innovation systems.

**FIGURE 1:**
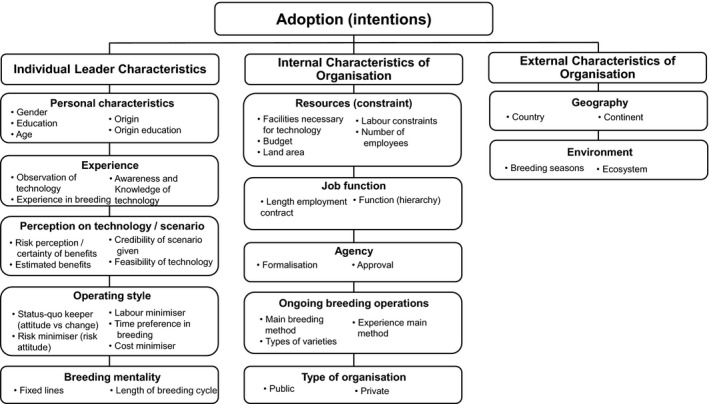
Framework for the adoption of new technologies in plant breeding. *Source*: Adapted from Lenaerts et al. (2018a)

Our first objective is to address the literature gap on technological change in crop breeding institutes and to formally examine technology adoption in plant breeding. Improving technology adoption upstream has significant multiplier effects on downstream impacts such that even small technological changes can add up to several billion US dollars of benefits accruing to farmers and consumers (Lenaerts et al., 2018b). Moving upstream has important theoretical and empirical implications. First, institutional factors such as the employment contract and the level of operational freedom increasingly play a role in technology adoption decisions when one moves from self‐employed actors (farmers) to employees operating in private or public organisations (breeders). To capture the diffusion of innovations in organisations, one needs to study both the organisation's internal and external characteristics and individual (leader) characteristics (Rogers, 2003). Lenaerts et al. (2018a) and the corresponding published dataset (Lenaerts et al., 2019b) adapted Rogers’ (2003) general organisational innovation framework to the case of technology adoption in plant breeding institutes (Figure 1). They conducted the first global online survey with 189 rice breeders from 51 rice‐growing countries worldwide to assess rice breeders’ and breeding institutes’ readiness to adopt alternative breeding methods. Whereas Lenaerts et al. (2018a) provide valuable descriptive information on breeders’ stated (that is, willingness or intention to adopt) and revealed (that is, actually implemented) adoption of accelerated breeding, more in‐depth insights into the complicated adoption process—from intentions to real behaviour—can be gained by analysing the data provided by Lenaerts et al. (2019b) through a more formal econometric approach. If institutional factors overrule individual preferences, revealed adoption models would deviate markedly from stated adoption models in the effects of individual breeder characteristics. For example, rice breeders with no intentions to adopt a technology may eventually do so because of managerial adoption decisions at the institutional level and not because of personal motivations. Understanding individual versus institutional adoption motivations is important since breeders in public breeding institutions are responsible for generating public goods. This top‐down view is in line with Rogers’ (2003, p. 402) statement that ‘an individual cannot adopt a new idea until an organisation [to which it belongs] has previously adopted it’. The reverse situation could hold when an individual breeder decides to adopt accelerated breeding before the technique becomes the norm within the company (thus acting as an innovation champion within the breeding institute (Rogers, 2003)). As a second objective, we contrast the determinants of stated and revealed adoption, in particular, to generate insights into the role of institutional factors in individual technology adoption decisions upstream in the innovation system.

Secondly, this article contributes some brief discussion on the estimation of multi‐stage models by comparing different approaches from the literature (Burke et al., 2015; Cappellari & Jenkins, 2006; Carreón & García, 2011; Cooper, 1997; Holm & Arendt, 2013).

Our article proceeds as follows. In the next section, we present some background on accelerated rice breeding. The second section presents our multi‐stage adoption model, while the third section discusses different econometric approaches to estimate multi‐stage models with a focus on three‐part models. We then briefly describe our variables and data. Next, we report and discuss our results and end by summarising our findings and reflecting on some policy implications.

## 2 BACKGROUND ON ACCELERATED RICE BREEDING

Accelerated rice breeding can be accomplished through various approaches, including molecular or conventional techniques (Forster et al., 2014). Although molecular breeding methods often lead to improved accuracy, cost or time savings, such methods are technically complex and expensive to set up. Therefore, rapid generation advance (RGA) is advocated as a feasible alternative for accelerating breeding in public‐sector breeding programmes in developing countries in the short term (Collard et al., 2017, 2019). RGA is a plant breeding technique first proposed by Goulden (1939) to address the problem of poor response to early generation selection due to high levels of heterozygosity. RGA essentially separates the selection for traits and breeding for homozygosity by taking only a single seed from each plant while progressing from one generation to the next (Collard et al., 2017). Since its development, RGA has been successfully implemented to breed different crops like barley, soybean and oats. However, it was only in 1963 that Nippon‐bare, the first rice variety bred by RGA, was released (Maruyama, 1989). Despite its early development, RGA adoption for rice breeding during the last two decades was shallow and limited mainly to Asia (Collard et al., 2017; Lenaerts et al., 2018a). Low adoption of RGA might be explained by breeding traditions rooted in low land and labour costs combined with little incentive in public breeding to adopt faster breeding. Despite low adoption rates, Lenaerts et al. (2018a) found rice breeders worldwide to be highly aware of RGA and its benefits, confident about the method itself and willing to adopt RGA. Yet, the lack of a greenhouse and no certainty about the benefits were the main constraints given by non‐adopters. We go beyond descriptive evidence and quantify the impact of different variables reflecting these hypothesised drivers of (non‐)adoption.

Compared to the conventional pedigree breeding method—which is still prevalent in rice breeding (Lenaerts et al., 2018a)—the advantages of RGA as a breeding method include technical simplicity, reduced operational costs (mainly land and labour) and time savings of around 1–3 years, depending on the number of growing seasons (Collard et al., 2017, 2019). Accelerating breeding is a proven method to increase the public benefits generated by breeding (Lenaerts et al., 2018b). Although RGA requires a greenhouse, it produces significant cost savings by lowering land and labour use intensities; the initial investment cost of a greenhouse repays itself within a few years. In terms of food security, the quicker release of new, improved varieties could help future generations escape lasting negative impacts from undernutrition such as stunting and mental impairment, improving their economic status (Lenaerts et al., 2019a)—assuming no bottlenecks in the dissemination of improved varieties downstream.

## 3 MULTI‐STAGE ADOPTION MODEL

**FIGURE 2:**
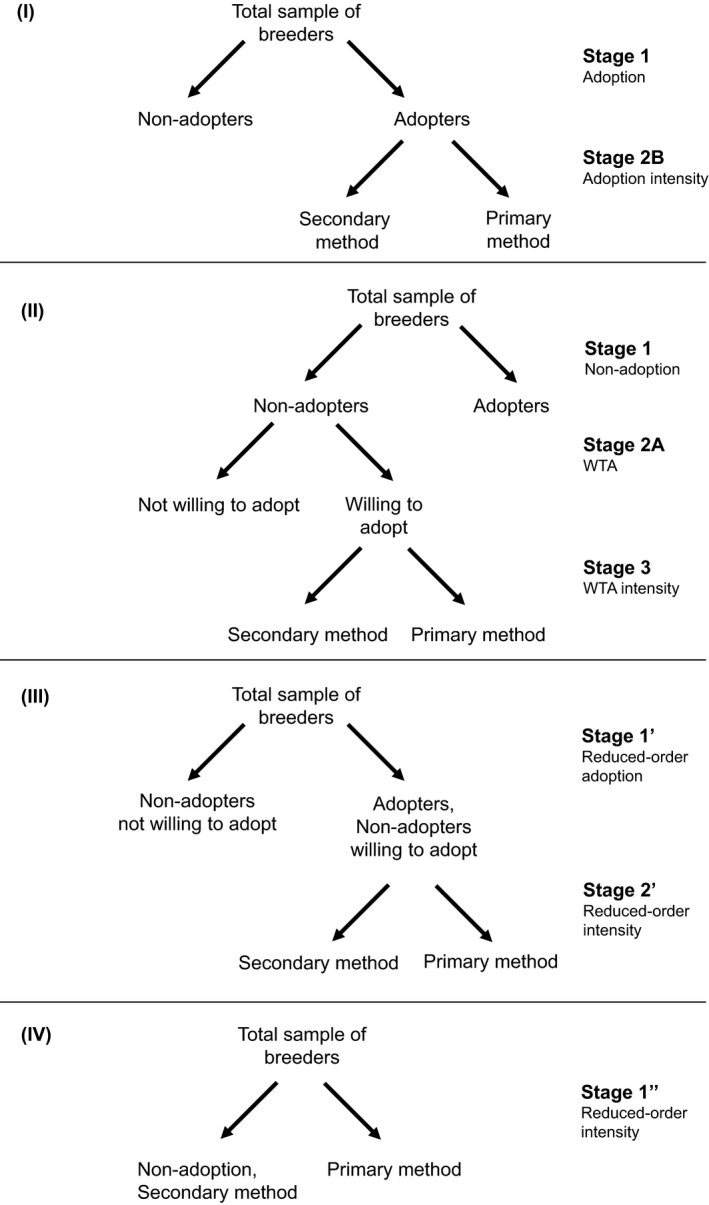
Graphic illustration of (I) two‐stage adoption model, (II) three‐stage willingness to adopt model, (III) reduced two‐stage adoption model, and (IV) reduced one‐stage adoption model

Given relatively low current adoption rates of accelerated rice breeding worldwide, Lenaerts et al. (2018a) and Lenaerts et al. (2019b) surveyed both revealed adoption and stated adoption intentions. This leads to a two‐stage model where the first or participation stage determines actual adoption (stage 1 in panels I and II in Figure 2), and the second stage determines adoption intentions for non‐adopters only (stage 2A in panel II).^1^ Positive revealed adoption and positive stated adoption intentions can be further decomposed into two levels of adoption intensity: (i) an ‘entry’ level where the technology is adopted as a secondary method; and (ii) an ‘advanced’ level where the technology is ‘mainstreamed’ as the primary method practised by the adopter in the adopting institute. The intensity of revealed adoption is captured by stage 2B in panel I of Figure 2 and the intensity of stated adoption by stage 3 in panel II.

For each breeder, we have binary information on different stages in the adoption pathway (that is, adoption, adoption intentions and the intensity of both adoption and adoption intentions). Because information on adoption intentions is only available for non‐adopters (and similarly for the intensity of adoption intentions), the stages differ in sample size. Figure 2 represents the stages considered in order of estimation (that is, from adoption over intention to intensity, or in descending order of sample size) (see the Appendix S1 for more details). Our econometric modelling approach visualised in Figure 2, however, cannot be interpreted as a sequential behavioural model; visual representations of the latter can be obtained through ‘adoption pathway analysis’, an approach recently developed by de Oca Munguia et al. (2021) to better represent and analyse the dynamics and diversity of adoption. Moreover, contrary to prevalent belief, multi‐stage model estimation cannot be used to infer sequential versus simultaneous decision‐making behaviour (Burke, 2019).

A three‐stage adoption model involving both real and stated behaviour is often reduced to a two‐stage model (a so‐called one‐way‐up model of adoption) by combining revealed adoption and stated adoption intentions (Cooper, 1997; Hubbell et al., 2000; Qaim & de Janvry, 2003). This reductionist approach, however, makes the implicit assumptions that (i) there is no selection present; (ii) actual users and intended users have the same utility function and associated coefficients (Cooper, 1997); and (iii) data on adoption intentions are not truly ‘missing’ for adopters as the latter can be assumed to be willing to adopt, which may not always hold, particularly in institutional settings where employees (e.g., breeders) report to management. The latter assumption reflects the premise that adopters are necessarily willing to adopt and people with adoption intentions have done so and are removed from our sample of people stating their adoption intentions—two assumptions that may not hold in upstream, institutional adoption settings. Advantages of the reduced model are increased efficiency in parameter estimation due to an increased number of observations (Hubbell et al., 2000), and less (hypothetical) bias from using stated instead of actual preferences (Cooper, 1997) at the cost of not fully exploiting the available information.

To indicate the bias introduced by the reductionist approach's assumptions, we estimate two reduced models. First, a two‐stage model is estimated where the participation stage distinguishes positive from negative adoption status (stated or revealed) (stage 1ʹ in panel III), and the outcome stage determines the adoption intensity for positive adoption levels (stage 2ʹ in panel III). The second models distinguishes positive from negative adoption status for RGA as primary method (stated or revealed) (stage 1ʹ in panel IV).

Finally, upstream in the innovation system, the first ‘adopt or not adopt’ stage in multi‐stage adoption decisions is increasingly governed by managerial and other institutional factors. As a result, we need to compare conditional and unconditional effects—that is, the effect on adoption intentions for non‐adopters, versus the effect for all sample members (Hoffmann & Kassouf, 2005). Since we are interested in understanding the fundamental drivers of upstream technology adoption (that is, representative of all breeders) instead of merely optimising an extension programme (that is, for non‐adopters), we will focus on unconditional rather than the commonly reported conditional effects.

## 4 MODEL ESTIMATION

Moving upstream in the innovation system inevitably leads to a smaller number of decision‐making units in the adoption process; from millions of farmers to hundreds of plant breeders operating under a few dozens of breeding institutes worldwide. Given this naturally low sample size (reflecting a limited population size) and evidence from simulation studies that instrumental variable analysis is only useful for either powerful instruments or an extensive sample (Boef et al., 2014; Crown et al., 2011), we refrain from such analysis and instead attempt to reduce endogeneity caused by omitted variable bias by including a broad set of covariates controlling for leader, institutional and external characteristics.

From a theoretical point of view, a Heckman selection model is appropriate when unobserved values are truly missing—that is, the latent value is unknown but might be observed under different circumstances—and we want to model potential behaviour rather than actual behaviour (Dow & Norton, 2003; Madden, 2008). In that case, measured behaviour is only capturing part of the population of interest, where both the outcome and selection process are correlated. A two‐part (subsample) model, on the other hand, looks at actual behaviour. Here, a zero outcome represents a utility maximising option.

In terms of estimation, the two‐part model is preferred under high collinearity^2^ due to its robustness, efficiency and simplicity to calculate, although its estimates will always be biased in the presence of selection (Bushway et al., 2007; Kennedy, 1998; Puhani, 2000). A small sample size (Hartman, 1991) and a lack of proper exclusion restrictions (Kennedy, 1998; Puhani, 2000) must be taken as a warning sign that collinearity might be problematic. Otherwise, the Heckman model can be estimated by the Heckit estimator (or Limited Information Maximum Likelihood, LIML)^3^ or the Full (Information) Maximum Likelihood estimator (FIML). Since the tri‐Heckit approach always represents a type of ‘forbidden regression’ and is never better than FIML, especially for small samples (Holm & Arendt, 2013), the FIML estimator should be attempted for three‐stage models, albeit at the risk of non‐convergence.

Inter‐stage correlation can be tested for using the likelihood‐ratio or a variable addition test relying on the original Heckit estimator, which has the advantage that no estimate of the information under the alternative hypothesis (unrestricted model) is required.^4^ Although they are technically not required for identification,^5^ it is more convincing to impose at least one within‐equation exclusion restriction for the second and third stages (Wooldridge, 2010, p. 702).

For three‐stage models, the literature offers two procedures to test for inter‐stage correlation (see the Appendix S1 for notation). A first approach is the full model, which assumes that correlation is possible between all stages without restrictions (Cappellari & Jenkins, 2006; Carreón & García, 2011; Holm & Arendt, 2013; Jensen et al., 2015; Maddala, 1983). Burke et al. (2015) propose a second, simplified approach, which assumes that any correlation between stages one and three can only occur through stage two.^6^

## 5 VARIABLES AND DATA

To study the case of multi‐stage technology adoption decisions upstream in the innovation system, we use data from a global online survey among rice breeders, featuring 158 cross‐sectional observations. The survey is described in detail in Lenaerts et al. (2018a), and the dataset is published and available online (Lenaerts et al., 2019b). The population of interest was carefully determined with help from the International Rice Research Institute and the Africa Rice Center and consists of active, non‐molecular rice breeders at national and international breeding institutes worldwide. Weighting adjustment was applied using the area of paddy rice harvested in 2015 to assess the sample's geographic representativeness. Only the share of Asian institutes shows signs of misrepresentation (we have a relatively low number of non‐Asian breeders, taking into account that 90% of rice production takes place in Asia). No outliers were removed. Breeders who used RGA only for testing were considered non‐adopters; breeders who used RGA as secondary or primary method were considered adopters.

Table 1 reports some descriptive statistics of the variables used in our model.^7^ Various individual leader characteristics were included in the analysis. The terms of the employment contract can affect both breeders’ risk and time preferences. First, indeterminate contracts provide more job security and could boost breeders’ confidence in adopting and experimenting with new, potentially more risky technologies. Second, under a fixed‐term contract, breeders may have less incentive to adopt technologies whose benefits do not accrue immediately. However, there is an increased urgency to deliver results within a fixed contract term, increasing interest in technologies that accelerate processes. Breeders’ perceptions were measured on several 7‐point scales. These variables were treated as continuous variables in the remainder of the analysis, since ordinal variables with at least five categories produce unbiased results, especially when the underlying concept is continuous and the intervals between points are approximately equal (Glass et al., 1972; Lubke & Muthén, 2004). Time preference was measured by asking breeders to what extent they consider the length of the breeding cycle to be an obstacle for improving farmers’ livelihoods (1 = not an obstacle, 7 = severe obstacle). Improving farmers’ livelihoods can be considered an implicit motivational incentive for public breeders (see below). Scores ranged from 3 to 6 with a median value of 5. Risk preference was measured as breeders’ stated likelihood of taking risks when choosing breeding methods (1 = avoids taking risks, 7 = likes taking risks) and ranged from 5 to 6 with a median value of 5. Awareness about the RGA breeding method was relatively high (88%), as was their stated credibility of the benefits of RGA (1 = not credible, 7 = very credible), which ranged from 5 to 7 with a median of 6. Credibility of benefits is the opposite of uncertainty about benefits, a driver of non‐adoption according to Lenaerts et al. (2018a). The benefits in question represented operational cost reductions as obtained by IRRI and were briefly described in a scenario included in the online survey (Lenaerts et al., 2018a). This realistic scenario with cost‐benefit estimates combined with follow‐up questions for corroboration helped reduce potential hypothetical bias in stated adoption intentions (sample of non‐adopters).

**TABLE 1:** Descriptive statistics for model variables

Variable	Description	Mean	SD
Adoption intentions (non‐adopters)			
WTA (*n* = 115)	1 = willing to adopt RGA (either as secondary or primary method); 0 = otherwise	0.74	0.44
WTA intensity (*n* = 85)	1 = willing to adopt RGA as primary method; 0 = willing to adopt RGA as secondary method	0.32	0.47
Revealed adoption			
Adoption (*n* = 158)	1 = adopted RGA (either as secondary or primary method); 0 = otherwise	0.27	0.45
Adoption intensity (*n* = 43)	1 = adopted RGA as primary method; 0 = adopted RGA as secondary method	0.19	0.39
Individual breeder characteristics			
Age	Years	45.72	9.31
Male	1 = male; 0 = female	0.82	0.39
PhD	1 = has PhD degree; 0 = otherwise	0.64	0.48
Indeterminate contract	1 = has indeterminate‐term contract; 0 = has fixed‐term contract	0.90	0.30
Time preference	1 = breeding cycle not an obstacle, …, 7 = severe obstacle	4.75	1.70
Risk preference	1 = avoid risk, …, 7 = like taking risks	5.16	1.54
Awareness	1 = aware of RGA; 0 = not aware	0.88	0.33
Credibility	1 = benefits of RGA not credible, …, 7 = very credible	5.47	1.41
Internal characteristics of the organisation			
Greenhouse	1 = greenhouse present; 0 = no greenhouse	0.70	0.46
Labour intensity	1 = above average; 0 = below average	0.27	0.45
Formalisation	1 = has opportunity to implement new techniques; 0 = otherwise	0.93	0.26
Inbred	1 = breeds inbred varieties only; 0 = otherwise	0.70	0.46
Private	1 = private institute; 0 = public institute	0.06	0.24
External characteristics of the organisation			
Asian	1 = institute located in Asia; 0 = otherwise	0.63	0.49
Seasons	Number of seasons (1, 2, 3)	1.62	0.55
Sample size *n*		158	

Notes

Lenaerts et al. (2018a) surveyed 189 rice breeders. However, due to missing values in the variables listed, a reduced dataset with 158 breeders was used instead. As a result, some of the descriptive statistics in Table 1 may differ from Lenaerts et al. (2018a).

Institutional characteristics include the presence of a greenhouse (70% of the cases)—the absence of which was a stated reason for non‐adoption (Lenaerts et al., 2018a)—whether the institute employing the breeder is private (that is, a commercial firm) (6% of the respondents) or public (94%) and the varieties that are being bred, that is, only inbreds (70%), only hybrids (7%) or both (23%). The dummy variable ‘private’ captures institutional differences that might affect adoption, such as the hypothesised lack of explicit incentives to adopt faster breeding in the public sector. The ‘level of formalisation’ was captured by asking breeders whether they have the opportunity to implement new techniques in their breeding method, and was relatively high (93% responded positively). This validates our framework's implicit assumption that breeders consider that they have sufficient agency to make adoption decisions. Labour intensity was measured as the ratio of seasonal workers employed during seeding and harvesting over the area of land used. To deal with extreme values and uneven distribution, we recoded above‐average values to one (27%) and zero otherwise (73%). Labour intensity captures the labour costs breeders face in their field operations, and hence their financial incentives for the adoption of labour‐saving technologies like RGA.

Finally, external characteristics of the organisation were captured through two variables, that is: (i) whether the institute is located in Asia (excluding the Middle East and Russia) (63%) or outside Asia (27%), since the majority of the world's rice is produced in Asia; and (ii) the number of breeding seasons per year (one, two or three), averaging 1.62, to capture the time‐specificity of the breeding programme.

## 6 RESULTS AND DISCUSSION

In this section, we first test the null hypothesis of no inter‐stage correlation and check the conditions of the selection test. We then present results for a three‐part probit model.

### 6.1 Model selection

A first step in testing for inter‐stage correlation is to identify exclusion restrictions in order to generate credible model estimates. When testing for inter‐stage correlation between adoption and adoption intentions, we imposed the exclusion restriction on age and age squared. Between adoption intentions and intended adoption intensity, we imposed the exclusion restriction on credibility. Between stated or revealed adoption and stated or revealed adoption intensity, we imposed the exclusion restriction on labour intensity. These variables are statistically significant in the selection equation and, if included, would not be significant in the outcome equation (Burke et al., 2015). To deal with the problem of separation or perfect prediction, we used the most common practical solution of omitting the offending variable from the analysis (Zorn, 2005). However, since the global population of rice breeders is limited—and correspondingly, any sample drawn from it—we attempted to exploit our dataset to the fullest extent possible. Therefore, despite the absence of estimates for the magnitude of the coefficients and standard errors, we treated cases of perfect predictability as valid evidence of correlation and reported and discussed the sign of the relationship in the remainder of the discussion accordingly.

Table 2 contains the condition index and the *p*‐values for the Heckit and LR test for inter‐stage correlation in our multi‐stage models, including the reduced models. For the three‐stage model, we included both the simplified (Burke et al., 2015) and full approach (Holm & Arendt, 2013). Given the exclusion restrictions, we found no significant correlation between adoption on the one hand, and adoption intention and adoption intensity on the other hand at the 5% level (stages 2A and 2B, respectively). For both the simplified and full approach, we found no correlation between adoption intention and adoption intention intensity (stages 2A and 3, respectively) using the Heckit test. The LR test becomes problematic as both the full and simplified approaches do not converge. Lastly, there is no evidence of any inter‐stage correlation for the reduced two‐stage model. Given our limited sample size, high collinearity levels, and lack of inter‐stage correlation (Table 2), we estimate all stages for the three‐stage model separately as a three‐part probit model.^8^

**TABLE 2:** Indices for inter‐stage correlation testing

Stages considered^a^	ρ	*n*	Condition index	*p*‐value Chi‐square test	*p*‐value LR‐test
Stages 1– 2B	ρ_12_	43	29	0.051	–^b^
Stages 1– 2A	ρ_12_	115	42	0.587	0.744
Stages 1– 2A– 3	ρ_13_, ρ_23_	85	44	0.226	–^b^
Stages 1– 2A– 3	ρ_23_	85	42	0.796	–^b^
Stages 1'– 2ʹ	ρ_1’2’_	128	59	0.325	0.544

Notes

*p*‐values based on robust standard errors.

^a^ See Figure 2.

^b^ Non‐convergence.

A possible explanation for the lack of selection are institutional enabling factors overruling individual drivers. We should not routinely expect that people with strong intentions to adopt are automatically shifted to the category of people stating their adoption intentions. Non‐adopters may have strong personal intentions to adopt but may be unable to do so because of lack of managerial support and freedom to implement and experiment with new techniques (Lenaerts et al., 2018a), an important enabling factor for technology adoption in the internal characteristics of an organisation.

### 6.2 Parameter estimates

The coefficient estimates of the two‐ and three‐part model are reported in Table 3 and the conditional and unconditional average marginal effects (AMEs) in Tables 4 and 5. The first and second data columns report the estimates for the determinants of the stated adoption intentions (stage 2A) and intended adoption intensities (stage 3) for the three‐part model, while the third and fourth data columns report the estimates for the determinants of the revealed adoption (stage 1) and adoption intensities of RGA (stage 2B) for the two‐part model. Tables 6 and 7 report the coefficients and conditional and unconditional average marginal effects of the reduced two‐part and one‐part adoption models.^9^ In the remainder of the analysis, we will juxtapose Tables 3, 4, 5, 6, 7 and discuss the results ‘horizontally’ by comparing the results, determinant per determinant, across different stages (see Figure 2), among different adoption categories (stated and revealed), among two levels of conditionality (conditional and unconditional) and between the full and reduced‐order forms. The conditional effect is the expected effect for those observed, whereas the unconditional effect represents the effect for all members of the sample (see the Appendix S1 for more details). We will pay particular attention to three types of inter‐stage, inter‐conditionality and inter‐model comparisons of the response curves of the determinants of the adoption models: (i) carry‐over, (ii) reversal, and (iii) bias. Carry‐over occurs when factors are significantly correlated with stated or revealed adoption across different stages. Reversal points to effects switching sign between stages. Bias refers to differences between the full and reduced‐order models.

**TABLE 3:** Coefficient estimates for three‐part willingness to adopt and two‐part adoption models

	Stated adoption intentions of non‐adopters		Revealed adoption by adopters	
	WTA	WTA intensity	Adoption	Adoption intensity
Individual breeder characteristics				
Age	0.092	–0.485*	0.322***	0.249
	(0.13)	(0.25)	(0.12)	(0.30)
Age squared	–0.001	0.006**	–0.003***	–0.003
	(0.00)	(0.00)	(0.00)	(0.00)
Male	–0.846*	–0.053	0.225	+^a^
	(0.45)	(0.50)	(0.38)	
PhD	0.243	–0.580	–0.321	–0.220
	(0.38)	(0.52)	(0.27)	(0.86)
Indeterminate contract	–0.191	+^b^	0.072	–2.627**
	(0.57)		(0.38)	(1.13)
Time preference	0.144	0.304**	–0.020	0.437**
	(0.09)	(0.12)	(0.06)	(0.19)
Risk preference	0.204*	0.571***	0.016	–0.184
	(0.10)	(0.15)	(0.08)	(0.17)
Awareness	0.743*	–1.438**	+^c^	+^c^
	(0.44)	(0.57)		
Credibility	0.245**	0.139	0.198**	1.072***
	(0.11)	(0.15)	(0.10)	(0.40)
Internal characteristics of the organisation				
Greenhouse	–0.372	–1.341***	0.638**	+^d^
	(0.35)	(0.47)	(0.26)	
Labour intensity	1.167***	–0.743	0.067	–2.612***
	(0.45)	(0.49)	(0.27)	(0.93)
Formalisation	1.658***	0.820	0.450	+^e^
	(0.51)	(0.59)	(0.63)	
Inbred	0.309	1.482**	0.512*	1.488
	(0.35)	(0.70)	(0.29)	(0.97)
Private	–1.662**	+^f^	1.490***	–^g^
	(0.75)		(0.51)	
External characteristics of the organisation				
Asian	–0.027	1.947***	–0.395	–1.446**
	(0.38)	(0.50)	(0.25)	(0.71)
Seasons	–0.600*	0.019	0.056	0.059
	(0.31)	(0.35)	(0.20)	(0.52)
Constant	–5.190*	3.359	–10.488***	–11.674
	(2.80)	(5.02)	(3.04)	(7.62)
*n*	115	85	158	43

Notes

Column 1 represents stage 2A, column 2 stage 3, column 3 stage 1 and column 4 stage 2B. Note that in stage 2A, non‐adopters were coded as 1 whereas the reverse is true for stage 2B. Robust standard errors in parentheses.

*p < 0.10; ** *p* < 0.05; *** *p* < 0.01.

^a^ Male = 1 predicts success perfectly.

^b^ Indeterminate contract = 1 predicts success perfectly.

^c^ Awareness = 1 predicts success perfectly.

^d^ Greenhouse = 1 predicts success perfectly.

^e^ Formalisation = 1 predicts success perfectly.

^f^ Private = 0 predicts failure perfectly.

^g^ Private = 0 predicts success perfectly.

**TABLE 4:** Conditional average marginal effects for three‐part willingness to adopt and two‐part adoption models

	Stated adoption intentions of non‐adopters		Revealed adoption by adopters	
	WTA	WTA intensity	Adoption	Adoption intensity
Individual breeder characteristics				
Age	0.004	0.004	0.003	–0.003
	(0.00)	(0.01)	(0.00)	(0.01)
Male	–0.165**	–0.010	0.061	+^a^
	(0.08)	(0.09)	(0.10)	
PhD	0.055	–0.112	–0.092	–0.035
	(0.09)	(0.10)	(0.08)	(0.14)
Indeterminate contract	–0.041	+^b^	0.020	–0.420***
	(0.12)		(0.10)	(0.11)
Time preference	0.032	0.058***	–0.006	0.070**
	(0.02)	(0.02)	(0.02)	(0.03)
Risk preference	0.045**	0.109***	0.004	–0.029
	(0.02)	(0.02)	(0.02)	(0.03)
Awareness	0.185	–0.290***	+^c^	+^c^
	(0.12)	(0.11)		
Credibility	0.055**	0.027	0.056**	0.171***
	(0.02)	(0.03)	(0.03)	(0.05)
Internal characteristics of the organisation				
Greenhouse	–0.078	–0.265***	0.169***	+^d^
	(0.07)	(0.08)	(0.06)	
Labour intensity	0.224***	–0.132*	0.019	–0.226***
	(0.07)	(0.08)	(0.08)	(0.07)
Formalisation	0.433***	0.144	0.113	+^e^
	(0.12)	(0.09)	(0.14)	
Inbred	0.071	0.226***	0.134*	0.189**
	(0.08)	(0.08)	(0.07)	(0.09)
Private	–0.440**	+^f^	0.465***	–^g^
	(0.19)		(0.13)	
External characteristics of the organisation				
Asian	–0.006	0.341***	–0.114	–0.214**
	(0.08)	(0.07)	(0.07)	(0.09)
Seasons	–0.134*	0.004	0.016	0.009
	(0.07)	(0.07)	(0.06)	(0.08)
*n*	115	85	158	43

Notes

Average Marginal Effects for variables are the discrete change from the base level. Robust standard errors in parentheses.

*p< 0.10; ** *p* < 0.05; *** *p* < 0.01.

^a^ Male = 1 predicts success perfectly.

^b^ Indeterminate contract = 1 predicts success perfectly.

^c^ Awareness = 1 predicts success perfectly.

^d^ Greenhouse = 1 predicts success perfectly.

^e^ Formalisation = 1 predicts success perfectly.

^f^ Private = 0 predicts failure perfectly.

^g^ Private = 0 predicts success perfectly.

**TABLE 5:** Unconditional Average Marginal Effects for Three‐Part Willingness to Adopt Adoption Model

	Stated adoption		Revealed adoption
	WTA	WTA intensity	Adoption intensity
Individual breeder characteristics			
Age	0.002	0.004	−0.001
	(0.01)	(0.01)	(0.02)
Male	−0.171	−0.052	0.011
	(0.11)	(0.08)	(0.04)
PhD	0.109	−0.023	−0.024
	(0.10)	(0.08)	(0.09)
Indeterminate contract	−0.045	−0.013	−0.097
	(0.14)	(0.04)	(0.10)
Time preference	0.027	0.037	0.013
	(0.03)	(0.03)	(0.06)
Risk preference	0.029	0.064*	−0.005
	(0.03)	(0.04)	(0.03)
Awareness	0.118	−0.096	+^a^
	(0.09)	(0.09)	
Credibility	−0.005	0.012	0.045
	(0.03)	(0.03)	(0.04)
Internal characteristics of the organisation			
Greenhouse	−0.192**	−0.206***	0.030
	(0.09)	(0.07)	(0.03)
Labour intensity	0.140	‐0.037	‐0.050
	(0.10)	(0.07)	(0.07)
Formalisation	0.263**	0.125**	0.021
	(0.12)	(0.06)	(0.04)
Inbred	−0.051	0.115*	0.056
	(0.09)	(0.06)	(0.06)
Private	−0.488***	−0.153***	0.068
	(0.1)	(0.04)	(0.07)
External characteristics of the organisation			
Asian	0.085	0.201***	−0.070
	(0.09)	(0.06)	(0.07)
Seasons	−0.108	−0.028	0.005
	(0.08)	(0.07)	(0.08)
*n*	115	85	43

Notes

Average Marginal Effects for dummy variables are the discrete change from the base level. Bootstrapped standard errors in parentheses.

*p < 0.10; ** *p* < 0.05; *** *p* < 0.01.

^a^ Awareness = 1 predicts success perfectly.

**TABLE 6:** Coefficient estimates for reduced two‐part and one‐part adoption models

	Reduced two‐part		Reduced one‐part
	Stated or revealed adoption	Stated or revealed adoption intensity	Stated or revealed adoption intensity
Individual breeder characteristics			
Age	0.193*	0.171	0.201*
	(0.10)	(0.14)	(0.11)
Age squared	−0.002*	−0.002	−0.002*
	(0.00)	(0.00)	(0.00)
Male	−0.654	0.311	0.173
	(0.42)	(0.35)	(0.33)
PhD	0.054	−0.641**	−0.499*
	(0.33)	(0.31)	(0.29)
Indeterminate contract	0.081	0.108	0.159
	(0.49)	(0.43)	(0.40)
Time preference	0.105	0.145**	0.145**
	(0.09)	(0.07)	(0.07)
Risk preference	0.168*	0.216**	0.209***
	(0.09)	(0.09)	(0.08)
Awareness	0.892**	−0.457	0.073
	(0.40)	(0.37)	(0.37)
Credibility	0.205**	0.112	0.144
	(0.09)	(0.11)	(0.10)
Internal characteristics of the organisation			
Greenhouse	−0.095	−0.090	−0.073
	(0.31)	(0.27)	(0.25)
Labour intensity	0.944**	−0.249	−0.074
	(0.39)	(0.28)	(0.25)
Formalisation	1.480***	0.639	0.922**
	(0.44)	(0.56)	(0.46)
Inbred	0.505*	0.697**	0.671**
	(0.30)	(0.32)	(0.27)
Private	−0.257	0.249	0.096
	(0.57)	(0.60)	(0.50)
External characteristics of the organisation			
Asian	−0.118	0.345	0.289
	(0.32)	(0.29)	(0.26)
Seasons	−0.504*	0.003	−0.115
	(0.26)	(0.23)	(0.22)
Constant	−7.200***	−7.324**	−9.047***
	(2.35)	(3.35)	(2.58)
*n*	158	128	158

Notes

Robust standard errors in parentheses.

*p < 0.10; **p < 0.05; ***p < 0.01.

**TABLE 7:** Conditional and unconditional average marginal effects for reduced two‐part and one‐part adoption models

	Conditional effects			Unconditional effects
	Reduced two‐part		Reduced one‐part	Reduced two‐part
	Stated or revealed adoption	Stated or revealed adoption intensity	Stated or revealed adoption intensity	Stated or revealed adoption intensity
Individual breeder characteristics				
Age	0.003	0.004	0.005	0.005
	(0.00)	(0.01)	(0.00)	(0.00)
Male	−0.111*	0.104	0.054	0.047
	(0.06)	(0.11)	(0.10)	(0.11)
PhD	0.011	−0.211**	−0.155*	−0.166*
	(0.06)	(0.09)	(0.09)	(0.09)
Indeterminate contract	0.016	0.036	0.049	0.035
	(0.10)	(0.14)	(0.12)	(0.12)
Time preference	0.021	0.049**	0.046**	0.047**
	(0.02)	(0.02)	(0.02)	(0.02)
Risk preference	0.033*	0.073***	0.066***	0.071***
	(0.02)	(0.03)	(0.02)	(0.03)
Awareness	0.215*	−0.152	0.023	−0.054
	(0.11)	(0.12)	(0.11)	(0.13)
Credibility	0.040**	0.037	0.045	0.046
	(0.02)	(0.03)	(0.03)	(0.03)
Internal characteristics of the organisation				
Greenhouse	−0.018	−0.031	−0.023	−0.032
	(0.06)	(0.09)	(0.08)	(0.08)
Labour intensity	0.158**	−0.084	−0.023	−0.013
	(0.05)	(0.09)	(0.08)	(0.09)
Formalisation	0.387***	0.204	0.251***	0.270***
	(0.12)	(0.16)	(0.10)	(0.10)
Inbred	0.104	0.227**	0.204***	0.218***
	(0.06)	(0.09)	(0.08)	(0.08)
Private	−0.054	0.083	0.030	0.041
	(0.13)	(0.20)	(0.16)	(0.16)
External characteristics of the organisation				
Asian	−0.023	0.116	0.090	0.084
	(0.06)	(0.10)	(0.08)	(0.09)
Seasons	−0.099*	0.001	−0.036	−0.038
	(0.05)	(0.08)	(0.07)	(0.07)
*n*	158	128	158	158

Notes

Average marginal effects for dummy variables are the discrete change from the base level. Robust standard errors for the conditional effects and bootstrapped standard errors for the unconditional effects in parentheses.

*p < 0.10; ** *p* < 0.05; *** *p* < 0.01.

When comparing Tables 3, 4, 5, 6, 7, we can make some general observations. We generally observe a heterogeneous mix of carry‐over and reversal between stated and revealed adoption in our multi‐stage model (Tables 3 and 4). However, as soon as the conditionality of positive adoption intentions is removed, all determinants in the revealed adoption stage become statistically insignificant, and almost none of the individual breeder characteristics in the adoption intention stage are carried over (Table 5). We further observe substantial deviations between our multi‐stage model and the reduced‐form models, providing insights into the bias generated by mixing stated and revealed adoption preferences (Tables 6 and 7). In contrast with our multi‐stage model, we see, for example, that the response curves detected by the reduced models are usually carried over from conditional to unconditional adoption.

The variable age features a U‐shaped relationship with the intended adoption intensity. However, this effect is not carried over to revealed adoption, where a statistically significant (1%) inverted U‐shaped relationship is found in the first adoption stage with a peak at around 45 years of age (Table 3). The inverse U‐shaped relationship is consistent with many adoption studies (e.g., Fernandez‐Cornejo, Hendricks & Mishra, 2005; Gine, Klonner & Finance, 2005) and is also captured by the reduced‐form two‐part and one‐part models. Female breeders are found to be 16.5% more likely to be willing to adopt RGA; however, this gender effect is reversed in the adoption stages (that is, all eight adopters who mainstreamed RGA as their primary method were male) (Tables 3 and 4). The unconditional average marginal effect is not significant; this suggests that if we consider the first adoption stage—which we hypothesise to be governed by institutional and managerial factors—adoption intentions of RGA are not expected to be different among all female or male breeders. The reduced two‐part model indicates that female breeders are 11.1% more likely to adopt, either through stated adoption intentions or revealed adoption (Table 7). A PhD degree is not a determining enabling factor in any of the adoption stages of our multi‐stage model (Tables 3, 4, 5). However, both reduced‐form models show how this factor is successfully carried over from conditional to unconditional adoption (intention) leading to the counterintuitive and potentially biased conclusion that a PhD degree hampers breeders in mainstreaming RGA (Tables 6 and 7).

Our empirical evidence suggests that job security through indeterminate contract terms boosts prospective users’ confidence in mainstreaming RGA as the primary method (that is, all 27 breeders willing to mainstream RGA as their primary method have an indeterminate contract) (Tables 3 and 4). However, this ambition was reversed in real adoption behaviour, where adopters with a fixed‐term contract were found to be 42% more likely to mainstream RGA, relative to breeders with a permanent contract (Tables 3 and 4). However, as soon as we remove the conditionality of adoption, the effect disappears in all stages (Table 5), suggesting that the contract terms are not a significant enabler of adoption for the full sample of breeders. The reduced‐form models do not pick up the role of the employment contract. Adoption intentions seem to be consistent with the behaviour predicted by the ‘reverse principal‐agent’ problem in public scientific research, in which, according to the Arrow and Lind (1978) theorem, the principal (research manager) is risk‐neutral and the agent (scientist) is risk‐averse (Bardsley, 1999). When eliciting their adoption ambitions, prospective adopters predictably responded like risk‐averse agents, whereas we observe the opposite in reality. The latter may suggest that the principal may have overruled real adoption decisions.

Risk and time preferences have often been found to be strong predictors of technology adoption (e.g., Bocqueho & Jacquet, 2010; Khanna, Louviere & Yang, 2017; Liu, 2013). Time preference is expected to drive the adoption of technologies that accelerate processes. Although adopters’ perception of urgency significantly increases the likelihood of mainstreaming of RGA in both adoption intensity stages by 5.8–7.0% per one‐point increase in the 7‐point scale of time preference (Tables 3 and 4), this effect disappears as soon as we remove the conditionality of adoption (Table 5). Reduced‐form models would have maintained that time preferences significantly drive mainstreaming of RGA at the rate of 4.6–4.9% per unit increase along the time preference scale, for both conditional and unconditional effects (Tables 6 and 7).

As expected, self‐identified risk‐takers express more ambitious adoption intentions and intensities. For every one‐point increase in the 7‐point scale of risk preferences, they are 4.5% more willing to adopt and 10.9% more willing to mainstream RGA—however, unconditionally, this driver mostly does not carry over (Tables 3, 4, 5). In the reduced‐form models the effect is carried over to all adoption stages, including the unconditional effects (Tables 6 and 7).

As expected, all breeders who have adopted RGA were aware of the technology. Although awareness has a significantly positive effect on breeders’ adoption intentions, breeders who were aware of RGA were more hesitant to mainstream the technology (Tables 3, 4, 5). This hesitance may suggest that awareness does not necessarily imply that breeders perceive the benefits of the technology to be credible enough to warrant full adoption. Therefore, we need to look at perceived credibility, which was the only factor that positively and significantly affects adoption throughout almost the entire adoption pathway, without becoming insignificant between stated and revealed adoption stages. Credibility was found to increase the likelihood of stated and revealed adoption by 5.5–5.6% and mainstreaming RGA by 17.1% per unit increase along the 7‐point scale (Tables 3 and 4). However, similarly to awareness, these effects disappear as soon as conditionality is removed (Table 5). This suggests that making individual breeders aware of the technology and demonstrating its credibility are not sufficient conditions for successful technology transfer of RGA worldwide; it might be more important that management is aware of the benefits of the technology in the first place. Reduced‐form models miss this reversal of awareness before adoption and would have concluded that the likelihood of adoption increases by 21.5% when breeders are made aware and by 4.0% for every unit increase in perceived credibility (Tables 6, 7).

A greenhouse is expected to be an essential enabling factor for RGA adoption (Collard et al., 2017). Indeed, greenhouse availability significantly predicts whether breeders have adopted and mainstreamed RGA; a greenhouse increases the likelihood of adoption by 16.9% and all eight adopters who have mainstreamed RGA are employed in a greenhouse‐endowed institute. However, this effect is reversed before adoption, as intended users with a greenhouse have a 26.5% lower probability to mainstream the technology. When we remove the conditionality of affirmative stated and revealed adoption intentions, the effect is not carried over to the revealed adoption stage. Moreover, the effect is reinforced in the adoption intention stage. We find that breeders in an institute with a greenhouse have a 19.2% lower likelihood to state willingness to adopt the technology and intended adopters have a 20.6% lower likelihood to intend to mainstream the technology. The unconditional adverse effects might point to the fact that institutes endowed with a greenhouse have relatively more financial resources and, therefore, would prefer more accurate but costly breeding methods (such as marker‐assisted breeding) over RGA. The reduced‐form models do not show significant effects of a greenhouse on (intended) adoption or adoption intensity (Tables 6 and 7).

Labour intensity significantly determines adoption intentions, which may be explained by the fact that RGA is a labour‐saving technology. Breeders deploying labour rates above the average are found to be 22.4% more likely to be willing to adopt RGA. Once RGA is adopted, lower labour intensity is associated with mainstreaming the technology, as adopters deploying below‐average labour rates are 22.6% more likely to mainstream RGA. These effects do not carry over to the first stage of revealed adoption (Tables 3 and 4) or any of the unconditional effects (Table 5), suggesting that labour‐saving may not have been breeders’ primary incentive for the adoption of RGA in 2015 when the survey was carried out and may not be the most potent argument in encouraging adoption of the technology. This preference may change in the future as labour costs are rising steeply in many rice‐growing countries, especially in Asia (ILO, 2018; Wiggins & Keats, 2014). The reduced two‐part model presents labour intensity as a driver of stated or revealed RGA adoption (conditional only), but not of adoption intensity (Tables 6 and 7).

The level of formalisation was found to affect all adoption stages positively, and the effect was significant in the adoption intention and revealed adoption intensity stages, where breeders with perceived opportunities for implementing new technologies are 43.3% more willing to adopt RGA and all eight adopters who mainstreamed the technology perceive to have the freedom to experiment. When we remove the conditionality, this agency effect persists in the adoption intention phase but disappears in the revealed adoption stage. The latter indicates a substantial number of breeders with positive adoption intentions and institutional flexibility who did not eventually adopt and were captured in the sample of stated adopters. This lack of adoption may imply that institutional flexibility is only an enabling factor and not a driver for RGA adoption and that other factors overrule it. In contrast, the reduced two‐part and one‐part models would have concluded that formalisation is a reliable driver, boosting the probability of adoption or mainstreaming RGA by 25.1%–38.7%.

A pure focus on inbred varieties tends to facilitate the adoption of RGA by 13.4% and mainstreaming of RGA by 18.9%–22.6% (Tables 3 and 4), which is consistent with hybrid rice breeding programmes lagging behind inbred programmes in terms of adoption and implementation of RGA in 2015.^10^ Once we remove the conditionality, the effect disappears in revealed adoption (Table 5). Both reduced two‐part and one‐part models would have concluded that inbred varieties are reliable drivers for adopting and mainstreaming RGA for both conditional and unconditional effects (Tables 6 and 7).

Since private breeding institutes are profit‐driven, it is not surprising to find 46.5% higher adoption rates of labour‐saving technologies like RGA in these institutes, although all eight breeders who have mainstreamed RGA as their primary method worked in public institutes (Tables 3 and 4). The results are reversed when moving from revealed adoption to adoption intentions (private breeders are 44% less willing to adopt RGA and all 58 breeders unwilling to mainstream RGA are public breeders) and from conditional to unconditional adoption intentions (Tables 4 and 5). An argument similar to the greenhouse effect applies here: private institutes might have relatively more financial resources and, therefore, prefer more accurate but costly breeding methods over RGA. Still, these results have to be interpreted with care as private breeders represent only a small minority (6%) of the total sample, as they were not explicitly targeted by the global survey (Lenaerts et al., 2018a).

Breeders operating in Asia were 34.1% more likely to express intentions of mainstreaming RGA. This effect is maintained (20.1%) when we remove conditionality—most likely due to the proximity to and visible presence of IRRI as a key adopter and promotor of accelerated breeding (Atlin et al., 2017; Collard et al., 2019). In reality, we observe significantly more (21.4%) mainstreaming of RGA outside Asia, but only conditionally (Tables 3, 4, 5).^11^ Since the adoption of RGA outside Asia is limited (Collard et al., 2017), this effect needs to be interpreted with caution.

Although the annual number of breeding seasons is not found to significantly predict adoption, we find that the higher the time‐specificity of the breeding programme, the more rice breeders express their interest in a technology that can accelerate rice breeding, RGA in this case (Tables 3 and 4). This effect is not carried over to the unconditional effects (Table 5) but is similarly captured in the reduced two‐part model (Tables 6 and 7).

## 7 CONCLUSION

Given future challenges related to climate change and the inadequacy of current crop yield trajectories to nourish the world's population by 2050, accelerated crop improvements are critical (Bailey‐Serres et al., 2019). Consequently, breeding institutes worldwide are currently considering redesigning and promoting the adoption of accelerated breeding in their breeding programmes. To provide policy advice in technology transfer from international to national agricultural research and extension systems (NARES), we move upstream in the agricultural innovation system and study the determinants of adoption of accelerated rice breeding using a published dataset from a survey of 158 rice breeders worldwide. Since the adoption of accelerated breeding is generally low, we consider both stated and revealed adoption decisions along the adoption pathway. We estimate a multi‐part (subsample) probit model that explicitly incorporates individual breeder characteristics and the organisation's internal and external characteristics, which is particularly important as breeders are involved with the creation of a public good. The lack of correlation between adoption and adoption intentions observed is consistent with adoption by institutions and managers rather than individual breeders. For comparison purposes, we also considered a reduced version of the three‐stage model, similar to Cooper (1997), and we examined the different results between the two approaches.

Juxtaposing the results among different adoption stages (stated intentions and revealed actions) and levels of conditionality (conditional and unconditional) generates useful insights in the role of institutional and managerial factors governing and overruling individual breeders’ adoption decisions. Comparing the results between our multi‐stage model and the reduced‐order models provides a measure of the magnitude of the bias that the latter would generate in the context of upstream technology adoption, caused by their strong assumptions and by contaminating revealed with stated adoption preferences, which could translate into misleading policy recommendations.

Reduced‐order models would have sent the signal to policy‐makers that individual breeders are sufficiently empowered such that their individual preferences can drive adoption decisions in rice breeding programmes worldwide. Our multi‐stage model does not confirm this evidence; that is, whereas individual breeder characteristics drive willingness to adopt the breeding technology for breeders who have not yet adopted, they do not seem to drive adoption intentions for the sample of breeders as a whole. Risk and time preferences could affect adoption more at management level than at the individual breeder level.

Regarding the organisation's internal characteristics, the presence of a greenhouse, breeders’ level of formalisation, and the private or public nature of the institute seem to matter most. Breeders who have the opportunity to implement new techniques are more willing to adopt accelerated breeding, which points to the importance of the managerial level in technology adoption by breeders. Breeders operating in institutes with a greenhouse and private institutes seem less interested in adopting accelerated breeding techniques. Budgetary differences can explain this as these institutes are likely to have substantial financial resources and, hence, might prefer more accurate but costly breeding methods over RGA. The reduced‐form models, however, do not pick up the greenhouse and private institute effect.

We do not find much evidence along the adoption pathway in support of the idea that the adoption of accelerated rice breeding is unconditionally determined by the organisation's external characteristics such as the location and proximity to technology promotors like IRRI and the time‐specificity of the breeding programme. In terms of policy implications, it means that there is no real evidence that structural hurdles prevent the transfer of accelerated rice breeding to research institutes worldwide, as long as the relevant decision‐makers in management are convinced of the technology's value.

Our results point towards the hypothesis that the adoption of accelerated breeding technologies by research organisations is influenced by institutional factors, not all of which are captured in our multi‐stage model. Our conclusion is that the decision still primarily lies with management. This management effect may explain the absence of correlation between the adoption and willingness to adopt stages, and the complete silence of our individual and external determinants when conditionality to the first decision stage is removed. The effect of managers making the final decision or even overruling their employees’ preferences and intentions can explain the lack of carry‐over in the models. The findings in our paper provide evidence of the potential bias in the context of upstream technology adoption originating from the omission of factors related to the organisation's internal and external characteristics and the use of reduced‐order models to capture behaviour along the adoption pathway.

If adoption decisions are ultimately made at the institutional and managerial level, technology transfer programmes should target institutions and managers, rather than direct users. In turn, managers could then survey and study what drives direct users’ adoption and mainstreaming intentions in their programmes to identify unconditional (rather than conditional) drivers, enablers, and barriers to adoption. In the case of adoption of accelerated breeding, our multi‐stage model suggests that managers should provide an enabling environment in which breeders are encouraged to take risks—an important factor—and are given sufficient freedom to experiment with and implement new technologies—a major enabler.

Future work should focus on the adoption pathway within organisations to further uncover the potential constraints or stimulants of the adoption of accelerated breeding. Moving from individual adoption, over between‐organisational adoption, towards the within‐organisational pathway follows the trend identified by Rogers (2003) in the diffusion of innovations literature. This final stage of the innovation process could be further uncovered through analysing organisational power structures and identifying potential frontrunners versus laggards of accelerated breeding (preferably over time) within an organisation. Although our survey questionnaire was designed to reduce hypothetical bias in stated adoption intentions, some bias may have remained and may have affected our results. Another limitation of this study is the exclusion of additional reasons for non‐adoption, such as cultural effects and breeding mindsets. Lastly, it is important not to overstate the impact of adopting RGA. Accelerated breeding is only one—albeit a significant—step upstream in the food system. Other areas of the innovation system that can benefit from acceleration are germplasm transfer and variety registration. Moreover, speed of varietal dissemination to farmers and breeders’ ability to incorporate real‐time producer, industry and consumer feedback into their product profiles are crucial factors that determine breeding programme success in improving food security (Custodio et al., 2016, 2019; Lenaerts et al., 2019a).

## Supporting information

Appendix S1

Appendix S2

## Footnotes

^1^
Our two‐stage model consists of a participation or selection equation determining the revealed adoption status, and an outcome equation determining the stated adoption status for those breeders who have not yet adopted. If the unobserved factors determining actual adoption and stated intentions are correlated—which might occur if breeders act as innovation champions, leaving the subsample of non‐adopters less willing to adopt alternative breeding methods—the (higher‐level) response equation determining stated intentions will be biased.

^2^
This refers to collinearity between the regressors in the outcome and selection equation, or equivalently, the correlation between the inverse Mills ratio and the other regressors in the outcome equation.

^3^
Despite the logical or ‘natural’ appeal of adding an inverse Mills ratio to a discrete outcome equation, there is no justification for this approach as a selection correction (Dubin and Rivers, 1989; Greene, 2012, p. 920; Terza, 2009; Wooldridge, 2010, pp. 596–597, 814). Wooldridge (2010) compares this approach to the so‐called forbidden regression from 2SLS estimation.

^4^
Again, although testing using correction terms such as the inverse Mills ratio is valid for detecting correlation involving discrete outcome equations, it is not a valid procedure to correct for selection bias (Wooldridge, 2010, p. 814).

^5^
In lieu of exclusion restrictions, identification relies on the nonlinearity of the inverse Mills ratio (Puhani, 2000).

^6^
Although this approach simplifies calculations considerably, it remains a strong assumption for which the authors provide no justification.

^7^
Because the dataset was reduced due to missing values, some of the descriptive statistics in Table 1 may not exactly match the ones reported by Lenaerts et al. (2018a).

^8^
We would like to thank an anonymous reviewer for pointing out that the lack of strong evidence of correlations across error terms should not be taken to weaken the case for multi‐stage estimation. Without conducting these tests, one would be in the dark regarding the biases and inconsistencies that might have been present.

^9^
Standard errors for the unconditional effects were calculated by bootstrapping following Burke (2009). We set the number of replications to 1000 (to end up with at least 618 successful ones) to obtain bootstrapped standard errors.

^10^
According to rice breeders, there are no real technical limitations for the adoption of RGA in hybrid rice programmes (Bert C.Y. Collard, Joshua N. Cobb and Jauhar Ali, personal communication).

^11^
One could argue that the fact that IRRI conducted the survey within its global rice breeding network (Lenaerts et al., 2018a) could have induced some social desirability bias.
