# Supplementary material for: Revisiting multi‐stage models for upstream technology adoption: Evidence from rapid generation advance in rice breeding
Source: J Agric Econ. 2021 Jul 4;73(1):277–300. doi: 10.1111/1477-9552.12450 (PMC9292170; doi:10.1111/1477-9552.12450)
Supplement: Supplementary file 1 — Appendix S1 [file JAGE-73-277-s001.pdf]

Revisiting multi-stage models for upstream technology adoption:

Evidence from rapid generation advance in rice breeding

Bert Lenaerts, Yann de Mey and Matty Demont

Online Appendix

Reproducibility

The data that support the findings of this study are openly available in Data in Brief at http://dx.doi.org/10.1016/j.dib.2019.103782. The Stata code to reproduce the results can be found in the Supplementary Information to this article.

Multi-stage specifications

The full set of adoption decisions can be represented as:

$$y_1 = y_1(\mathbf{X},\mathbf{Y},\mathbf{Z}), \text{ for all breeders} \quad (1)$$

$$y_2^A = y_2^A(\mathbf{X},\mathbf{Y},\mathbf{Z}), \text{ for non-adopters} \quad (2)$$

$$y_3 = y_3(\mathbf{X},\mathbf{Y},\mathbf{Z}), \text{ for non-adopters willing to adopt, and} \quad (3)$$

$$y_2^B = y_2^B(\mathbf{X},\mathbf{Y},\mathbf{Z}), \text{ for adopters} \quad (4)$$

where y_1 is a binary indicator of whether the breeder has adopted the technology, y_2^A is a binary indicator of the adoption intention, y_2^B and y_3 are binary indicators of adoption intensity and **X**, **Y** and **Z** are vectors representing individual breeder characteristics and breeding institutes' internal and external characteristics, respectively.

The reduced set of adoption decisions can be represented as:

$$y_1^R = y_1^R(\mathbf{X},\mathbf{Y},\mathbf{Z}), \text{ for all breeders} \quad (5)$$

$$y_2^R = y_2^R(\mathbf{X},\mathbf{Y},\mathbf{Z}), \text{ for adopters and non-adopters willing to adopt, and} \quad (6)$$

$$y^R = y^R(\mathbf{X},\mathbf{Y},\mathbf{Z}), \text{ for all breeders} \quad (7)$$

where y_1^R is a binary indicator of whether the breeder has adopted or is willing to adopt the technology, and y_2^R and y^R are indicators of whether the breeder has adopted or is willing to adopt the technology as a secondary or primary method.

Full three-stage Heckman selection model

To remain as general as possible, we present the full three-stage Heckman selection model with different regressors for the selection (or participation) and outcome equations. The three-part model is simply this model with zero correlations.

First stage	Second stage	Third stage
$y_j^* = \mathbf{x}_{1j}\boldsymbol{\beta}_1 + u_{1j}$	$y_j^{**} = \mathbf{x}_{2j}\boldsymbol{\beta}_2 + u_{2j}$	$y_j^{***} = \mathbf{x}_{3j}\boldsymbol{\beta}_3 + \mathrm{u}_{3j}$
$y_j^{\text{first}} = 1 \text{ if } y_j^* > 0$	$y_j^{\text{second}} = 1 \text{ if } y_j^{**} > 0$	$y_j^{\text{third}} = 1 \text{ if } y_j^{***} > 0$
$y_j^{\text{first}} = 0 \text{ if } y_j^* \leq 0$	$y_j^{\text{second}} = 0 \text{ if } y_j^{**} \leq 0$	$y_j^{\text{third}} = 0 \text{ if } y_j^{***} \leq 0$

where y_j^{third} is only observed if $y_j^{\text{second}} = 1$ and y_j^{second} is only observed if $y_j^{\text{first}} = 1$. In this set of equations, the index j represents the jth observation in the dataset of breeders; $\mathbf{x}_1$, $\mathbf{x}_2$ and $\mathbf{x}_3$ are the independent variables determining the first, second and third stage respectively; $\boldsymbol{\beta}_1$, $\boldsymbol{\beta}_2$ and $\boldsymbol{\beta}_3$ are the vectors of parameters to be estimated. The latent variables y_j^*, y_j^{**} and y_j^{***} are typically unobserved. We assume trivariate normality:

$$\mathbf{u}_j = \begin{bmatrix} u_{1j} \\ u_{2j} \\ u_{3j} \end{bmatrix} \sim N\left(\begin{bmatrix} 0 \\ 0 \\ 0 \end{bmatrix}, \begin{bmatrix} 1 & \rho_{12} & \rho_{13} \\ \rho_{12} & 1 & \rho_{23} \\ \rho_{13} & \rho_{23} & 1 \end{bmatrix} \right) \quad \text{(A1)}$$

where ρ_{12}, ρ_{13} and ρ_{23} are the correction terms representing the correlation between the error terms.

Furthermore, $\mathbf{y}_j = \begin{bmatrix} y_j^{\text{first}} \\ y_j^{\text{second}} \\ y_j^{\text{third}} \end{bmatrix}$, and $\boldsymbol{\rho} = \begin{bmatrix} \rho_{12} \\ \rho_{13} \\ \rho_{23} \end{bmatrix}$.

Likelihood functions

For any given observation, i, the tri-probit Heckman selection model has the following likelihood function:

$$\mathrm{L}(\mathbf{y}|\boldsymbol{\beta},\boldsymbol{\rho}) = \prod_{j=1}^{n} \left[1-\Phi(\mathbf{x}_{1j}\boldsymbol{\beta}_1)\right]^{1-y_j^{\text{first}}}$$

$$\times \left[\Phi\left(\mathbf{x}_{1j}\boldsymbol{\beta}_1\right) \left\{ \begin{matrix} \left(1-\Phi(\mathbf{x}_{2j}\boldsymbol{\beta}_2,\rho_{12})\right)^{1-y_j^{\text{second}}} \\ \left(\Phi\left(\mathbf{x}_{2j}\boldsymbol{\beta}_2,\rho_{12}\right) \left(1-\Phi\left(\mathbf{x}_{3j}\boldsymbol{\beta}_3,\rho_{13},\rho_{23}\right)\right)^{1-y_j^{\text{third}}} \right)^{y_j^{\text{second}}} \\ \left(\Phi(\mathbf{x}_{3j}\boldsymbol{\beta}_3,\rho_{13},\rho_{23})\right)^{y_j^{\text{third}}} \end{matrix} \right\} \right]^{y_j^{\text{first}}} \quad \text{(A2)}$$

where Φ is the standard normal cumulative distribution function.

Holm and Arendt (2013) and Maddala (1983) provide the following general formulation for a (full) tri-probit model:

$$\Pr\left(y^{\text{third}}=1|y^{\text{second}}=1,y^{\text{first}}=1,\mathbf{x}_1,\mathbf{x}_2,\mathbf{x}_3\right)=\Phi\left(\mathbf{x}_3\boldsymbol{\beta}_3-\rho_{23}\mathrm{M}_{21}-\rho_{13}\mathrm{M}_{12}\right) \tag{A3}$$

$$\mathrm{M}_{ij}=\left(\mathrm{P}_i-\rho_{12}\mathrm{P}_j\right)/\left(1-\rho_{12}^2\right) \tag{A4}$$

$$\mathrm{P}_2=\{-\varphi(-\boldsymbol{x}_2\boldsymbol{\beta}_2)[1-\Phi(r_{12}(-\boldsymbol{x}_1\boldsymbol{\beta}_1+\rho_{12}\boldsymbol{x}_2\boldsymbol{\beta}_2))]$$

$$-\rho_{12}\varphi(-\boldsymbol{x}_1\boldsymbol{\beta}_1)[1-\Phi(r_{12}(-\boldsymbol{x}_2\boldsymbol{\beta}_2+\rho_{12}\boldsymbol{x}_1\boldsymbol{\beta}_1))]\}/\Phi_2\left(\mathbf{x}_2\boldsymbol{\beta}_2,\mathbf{x}_1\boldsymbol{\beta}_1;\rho_{12}\right) \tag{A5}$$

where $r_{12}=1/\sqrt{1-\rho_{12}^2}$, φ is the standard normal probability density function, Φ_2 is the standard normal cumulative bivariate distribution function and P_1 is obtained by interchanging $\mathbf{x}_2\boldsymbol{\beta}_2$ by $\mathbf{x}_1\boldsymbol{\beta}_1$.

Formulations for a tri-probit model using the simplified approach proposed by Burke et al. (2015) can be represented as:

$$\Pr\left(y^{\text{third}}=1|y^{\text{second}}=1,y^{\text{first}}=1,\mathbf{x}_1,\mathbf{x}_2,\mathbf{x}_3\right)$$

$$=\Phi\left(\mathbf{x}_3\boldsymbol{\beta}_3+\rho_{23}\,\varphi\left[\mathbf{x}_2\boldsymbol{\beta}_2+\rho_{12}\frac{\varphi(\mathbf{x}_1\boldsymbol{\beta}_1)}{\Phi(\mathbf{x}_1\boldsymbol{\beta}_1)}\right]\Big/\Phi\left[\mathbf{x}_2\boldsymbol{\beta}_2+\rho_{12}\frac{\varphi(\mathbf{x}_1\boldsymbol{\beta}_1)}{\Phi(\mathbf{x}_1\boldsymbol{\beta}_1)}\right]\right) \tag{A6}$$

(Un)conditional effects for three-part probit models

Even when certain parameters can be calculated separately, the overall effect of a given independent variable should always be determined by considering all respective parameters together (Burke, 2009). Therefore, we need to calculate the conditional and unconditional (marginal) effects (Hoffmann and Kassouf, 2005).[1] The conditional effect is the expected effect conditional on being observed. The unconditional effect for a two-stage model consists of two parts: the effect associated with a change in the probability of being observed, and the effect associated with a change in outcome for those observed (Hoffmann and Kassouf, 2005). For a three-part probit model, the conditional expected value, where x_j is a continuous variable that can belong to $\mathbf{x}_1$, $\mathbf{x}_2$ and $\mathbf{x}_3$, can be represented as:

$$\Pr\left(y^{\text{third}}=1|y^{\text{second}}=1,y^{\text{first}}=1,\mathbf{x}_3\right)=\Phi(\mathbf{x}_3\boldsymbol{\beta}_3), \tag{A7}$$

with its marginal effect:

$$\partial\Pr\left(y^{\text{third}}=1|y^{\text{second}}=1,y^{\text{first}}=1,\mathbf{x}_3\right)/\partial\mathbf{x}_j=\varphi(\mathbf{x}_3\boldsymbol{\beta}_3)\times\beta_{3j}. \tag{A8}$$

The unconditional expected value can be represented as:

[1] For binary outcome variables, it is more meaningful to talk about conditional and unconditional expected probabilities rather than mean values.

$$\Pr\left(y^{\text{third}}=1\,\middle|\,\mathbf{x}_1,\mathbf{x}_2,\mathbf{x}_3\right)=\Phi(\mathbf{x}_1\boldsymbol{\beta}_1)\times\Phi(\mathbf{x}_2\boldsymbol{\beta}_2)\times\Phi(\mathbf{x}_3\boldsymbol{\beta}_3), \tag{A9}$$

with its marginal effect:

$$\partial\Pr\left(y^{\text{third}}=1\,\middle|\,\mathbf{x}_1,\mathbf{x}_2,\mathbf{x}_3\right)/\partial x_j=\varphi(\mathbf{x}_1\boldsymbol{\beta}_1)\times\beta_{1j}\times\Phi(\mathbf{x}_2\boldsymbol{\beta}_2)\times\Phi(\mathbf{x}_3\boldsymbol{\beta}_3)$$

$$+\Phi(\mathbf{x}_1\boldsymbol{\beta}_1)\times\varphi(\mathbf{x}_2\boldsymbol{\beta}_2)\times\beta_{2j}\times\Phi(\mathbf{x}_3\boldsymbol{\beta}_3)+\Phi(\mathbf{x}_1\boldsymbol{\beta}_1)\times\Phi(\mathbf{x}_2\boldsymbol{\beta}_2)\times\varphi(\mathbf{x}_3\boldsymbol{\beta}_3)\times\beta_{3j}. \tag{A10}$$

If $\mathbf{x}_j$ is not part of $\mathbf{x}_1$, $\mathbf{x}_2$ or $\mathbf{x}_3$, then β_{1j}, β_{2j}, or β_{3j}, respectively, are zero, and specific terms may drop.

References

Burke, W. J. 2009. Fitting and interpreting Cragg's tobit alternative using Stata, *Stata Journal*, vol. 9, no. 4, 584

Burke, W. J., Myers, R. J., and Jayne, T. S. 2015. A Triple-Hurdle Model of Production and Market Participation in Kenya's Dairy Market, *American Journal of Agricultural Economics*, vol. 97, no. 4, 1227–46

Hoffmann, R. and Kassouf, A. L. 2005. Deriving conditional and unconditional marginal effects in log earnings equations estimated by Heckman's procedure, *Applied Economics*, vol. 37, no. 11, 1303–11

Holm, A. and Arendt, J. N. 2013. Evaluating the performance of simple estimators for probit models with two dummy endogenous regressors, *Journal of Statistical Computation and Simulation*, vol. 83, no. 6, 1156–78

Maddala, G. S. 1983. Models with self-selectivity, pp. 257–91, in *Limited-dependent and qualitative variables in econometrics*, Cambridge University Press, New York, USA
